# From Affective Science to Psychiatric Disorder: Ontology as a Semantic Bridge

**DOI:** 10.3389/fpsyt.2018.00487

**Published:** 2018-10-08

**Authors:** Rasmus Rosenberg Larsen, Janna Hastings

**Affiliations:** ^1^Department of Philosophy and Forensic Science Program, University of Toronto, Mississauga, ON, Canada; ^2^Department of Biological Sciences, Babraham Institute, University of Cambridge, Cambridge, United Kingdom

**Keywords:** affective science, psychiatry, RDoC, DSM, ontology, data science, integration

## Abstract

Advances in emotion and affective science have yet to translate routinely into psychiatric research and practice. This is unfortunate since emotion and affect are fundamental components of many psychiatric conditions. Rectifying this lack of interdisciplinary integration could thus be a potential avenue for improving psychiatric diagnosis and treatment. In this contribution, we propose and discuss an ontological framework for explicitly capturing the complex interrelations between affective entities and psychiatric disorders, in order to facilitate mapping and integration between affective science and psychiatric diagnostics. We build on and enhance the categorisation of emotion, affect and mood within the previously developed Emotion Ontology, and that of psychiatric disorders in the Mental Disease Ontology. This effort further draws on developments in formal ontology regarding the distinction between normal and abnormal in order to formalize the interconnections. This operational semantic framework is relevant for applications including clarifying psychiatric diagnostic categories, clinical information systems, and the integration and translation of research results across disciplines.

## 1. Introduction

Emotion, affect, and mood are a central aspect of diagnosis, treatment, and research into psychiatric disorders. It is the engulfing experience of fear that is central to what we term phobia, persisting sadness and depleted affect characterize depression, sporadic outbursts of anger are focal to intermittent explosive disorder, and so on. Yet, research results from affective science have been slow to translate into psychiatry (1), and studies investigating the relationships between affective and psychiatric phenomena, e.g., sadness and depression, are significantly outnumbered by studies dealing with the individual categories in isolation (2).

There are significant theoretical and practical obstacles to conducting research that addresses questions across two historically separate domains such as affective science and psychiatry (3, 4). However, there is a growing community-wide recognition that the standard practices of research in isolated disciplines and diagnostic categories have not led to sufficient progress in relieving the burden of psychiatric disorder, and thus that a new framework to enable such integration is both urgent and necessary (5).

Semantic frameworks structure research in a domain by picking out the types of entities that are believed to be relevant for research and practice, such as types of emotion in affective science and types of psychiatric disorder in psychiatry. The adoption of a particular semantic framework in a given field may have far-reaching consequences, such as for the allocation of research funding and the determination of legal and ethical matters in the sociological environment. Historically, psychiatry has largely been structured according to the diagnostic kinds formalized in the various editions of the Diagnostic and Statistical Manual (DSM) and the International Classification of Disorders (ICD), while more recently the Research Domain Criteria (RDoC) was introduced (6). RDoC is explicitly multi-domain and integrative in its design (7, 8), re-organizing research efforts into upper-level traits (e.g., negative valence) and cross-cutting constructs, instead of the traditional diagnostic categories. It was anticipated that re-directing research efforts into a shared framework of upper-level traits might facilitate a more efficient integration of knowledge discovery across all the relevant sciences (6) and better reflect dimensionality in applicable phenomena (9–11). However, the RDoC proposal has had a mixed reception in its current form, being criticized for failing to adequately address the challenges it was designed for, while moreover introducing other problems, such as a lack of construct validity and a disconnect from clinical relevance (12–15).

The need to rethink the semantic framework of psychiatry in order to enable cross-disciplinary translation and integration has thus still not been adequately addressed (16, 17). The community is embarking on an active process of taxonomic evolution, including the development of a hierarchical taxonomy of psychopathology as another alternative (18, 19) and data-sharing initiatives (20). The challenges posed by this situation are both practical and theoretical: practical, insofar as it requires ongoing collaborative work to agree on a shared semantic framework between multiple domains (21), and theoretical, because there are significant conceptual hurdles involved in developing a semantic framework with enough substance to accommodate the complexities of each domain, across not only psychiatry and neuroscience, but the full range of biological and human sciences in a comprehensive “multilevel, systemic approach” (22).

In this paper, we propose that a framework based on *applied ontologies* can serve as a practical aid to facilitate the needed conceptual integration and stabilization of research constructs in this rapidly evolving focus area. The framework using applied ontologies offers not a brand-new taxonomy, but a method to integrate between different, perhaps competing and contradictory, taxonomies, and to connect the taxonomies thus integrated to the actual data that arises from research, in such a fashion that empirical results (arising from research conducted across different semantic frameworks including the DSM) can be used to inform the further development of the taxonomies.

Our approach will be developed in an outline illustrated by examples taken from affective science and psychiatry. First, we sketch the methodological framework we propose as a viable solution to the aforementioned problems. Secondly, we give an outline of how this methodological framework can be applied to build semantic bridges between affective science and the psychiatric domain. We conclude by discussing central limitations inherent to our methodology.

## 2. Background: applied ontology

**Figure 1:**
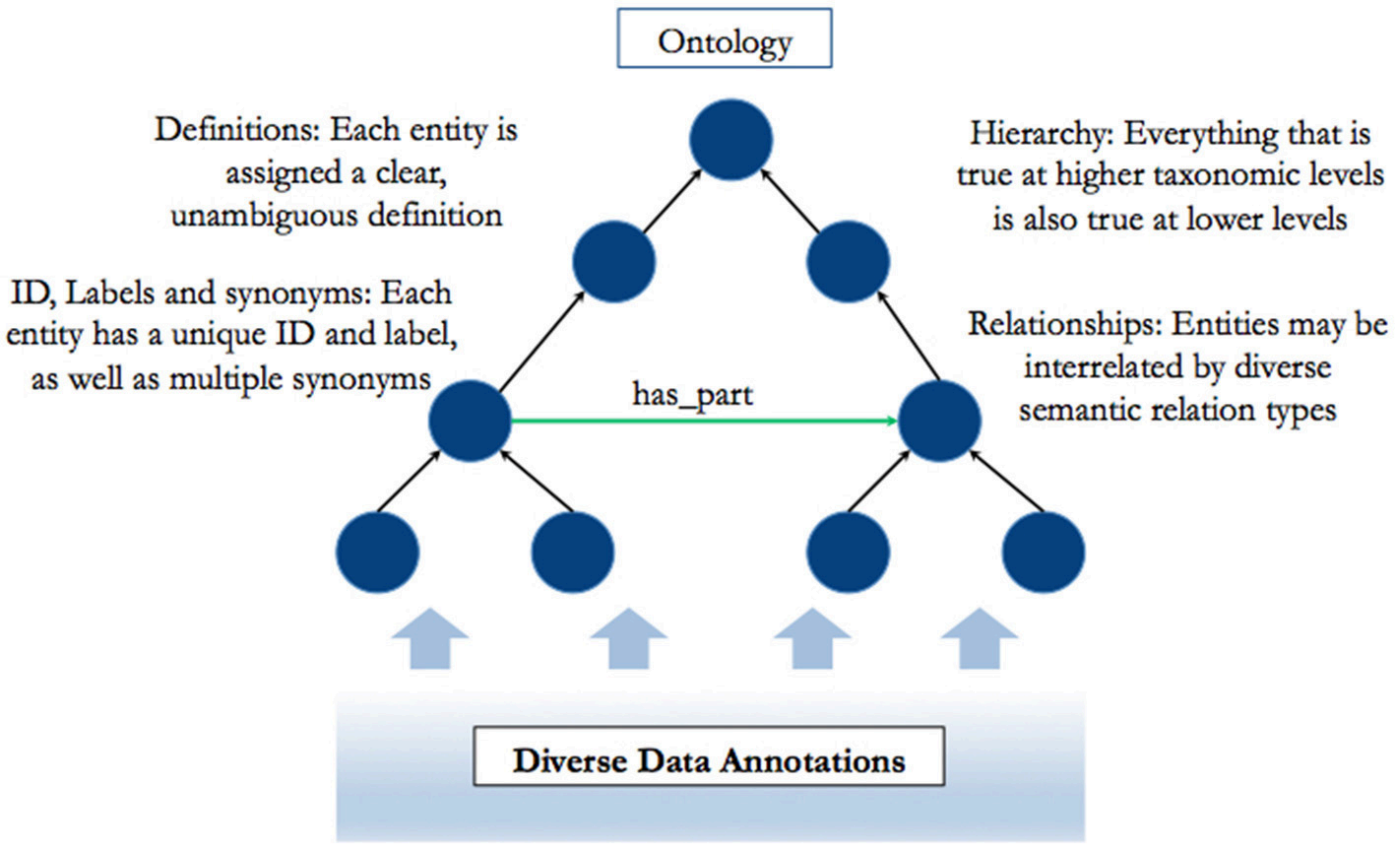
An overview of the nature and structure of an applied ontology.

In recent years, research across scientific domains has been increasingly characterized by a simultaneous shift toward unmanageable quantities of data (“big data”) and a raised awareness of the importance of conceptual integration across perspectives, theories, and disciplinary boundaries. In this context, applied ontologies have emerged as a tool to structure and organize data, and enable conceptual integration (23). Applied ontologies consist of a set of clearly defined entities (which may, however, each have multiple labels), structured hierarchically, and interconnected by defined relations (see Figure 1).

An example of such an ontology is the Gene Ontology (GO) (24), which currently (as of May 2018) contains 49,495 entities interconnected by more than 90,000 relations, widely and successfully applied across many different aspects of research in the biological sciences [e.g., (25)]. The success of the GO has spawned similar standardization efforts across and between a diverse range of other subject areas such as phenotypes, medical conditions, chemicals, cells, engineering, sociology, etc., many of which are accessible as open-source ontologies in the Open Biological and Biomedical Ontology Foundry (OBO) (26). These ontologies enable structured and harmonized annotation of data, mitigating the challenge of ever-expanding research output through a clever use of digital “data science” technologies (23), and thereby facilitating both the analysis of raw research data and the mutual integration of findings across domains and subject matters (27). An applied ontology is an externalized (computationally embedded) understanding of the nature of entities in the world, as viewed through the perspectival lens of a specific field of research and practice (28). It forms a part of the research process itself insofar as computational tools (such as data mining, analysis and aggregation) form a part of the research process and harness the ontology for their operation. And at the same time, the ontology forms a part of the community evolution of understanding about the nature of entities in the field: ontologies serve as structures which capture the process and outcome of debates within the field, helping to facilitate the stabilization and manageability of discourses [see e.g., (29) for elaboration on the need for construct stabilization in psychiatry].

While applied ontologies generally reflect the subject matter of a single domain, they have also come to be extended into concrete bridges between different domains. So there is, for instance, an ontology representing biological processes (the aforementioned GO) and another representing chemical entities [Chemical Entities of Biological Interest, or ChEBI; (30)]. From here, so-called “bridging statements” in the form of inter-ontology relationships crossing between two different domains can be created, for example, by representing the ways in which different chemicals participate in, and contribute to biological processes [e.g., (31)]. These bridging statements capture ontological knowledge, although not about one domain or another, but rather the ways in which the entities between two different domains relate to each other. In other words, bridging statements define lines along which interdisciplinary translation and integration may proceed.

It is precisely such an interdisciplinary effort that we offer in this paper. We aim to show, in outline, how entities from an affective science ontology (the Emotion Ontology) can be connected via bridging statements to entities in an ontology for psychiatric disorders (the Mental Disease Ontology), in such a fashion that the bridges enable integration and translation, yet remain agnostic to the debates and research paradigms within each of these domains.

Before we can turn to concrete examples of how we propose to execute this semantic bridging, we need first to briefly introduce the constellation of ontologies between which we are seeking to construct these bridges.

### 2.1. The mental functioning ontology (MF)

Moving beyond traditional disorder categories in psychiatry necessitates a greater focus on the symptoms and phenomenology of mental experiences.

**Figure 2:**
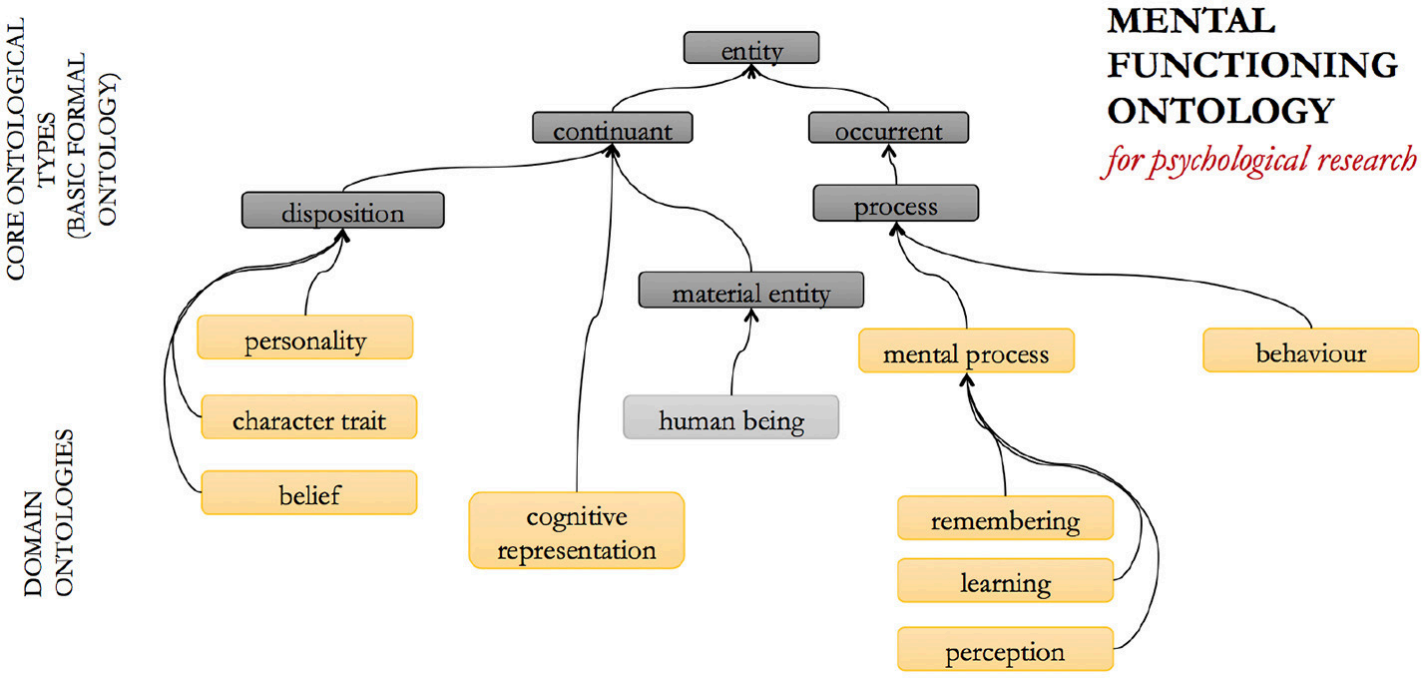
The mental functioning (MF) ontology: selected entities and relations.

The Mental Functioning Ontology (MF, Figure 2) represents all aspects of “ordinary” mental functioning and phenomenology that are not explicitly affective or psychiatric in their nature (32, 33). It includes, for example, entities such as consciousness, perception, thinking, and believing, and emphasizes the first-person and experiential perspective of human mental functioning. It also serves as a mid-level ontology for the whole of the psychological domain and is thus re-used modularly within the other ontologies in this suite.

### 2.2. The emotion ontology (EM)

The Emotion Ontology (EM) was developed for the domain of affective science (34). The entities it categorizes and defines include emotions, moods, and varying related entities such as emotional behavior, facial expressions, subjective feelings, etc., and the dimensions along which affective experience may be categorized, such as valence and arousal. The EM was developed with the view that use of the term “emotion” by itself has multiple meanings, and the EM therefore offers a way of exhaustively rendering these potential ambiguities explicit in a non-ambiguous semantic framework.

**Figure 3:**
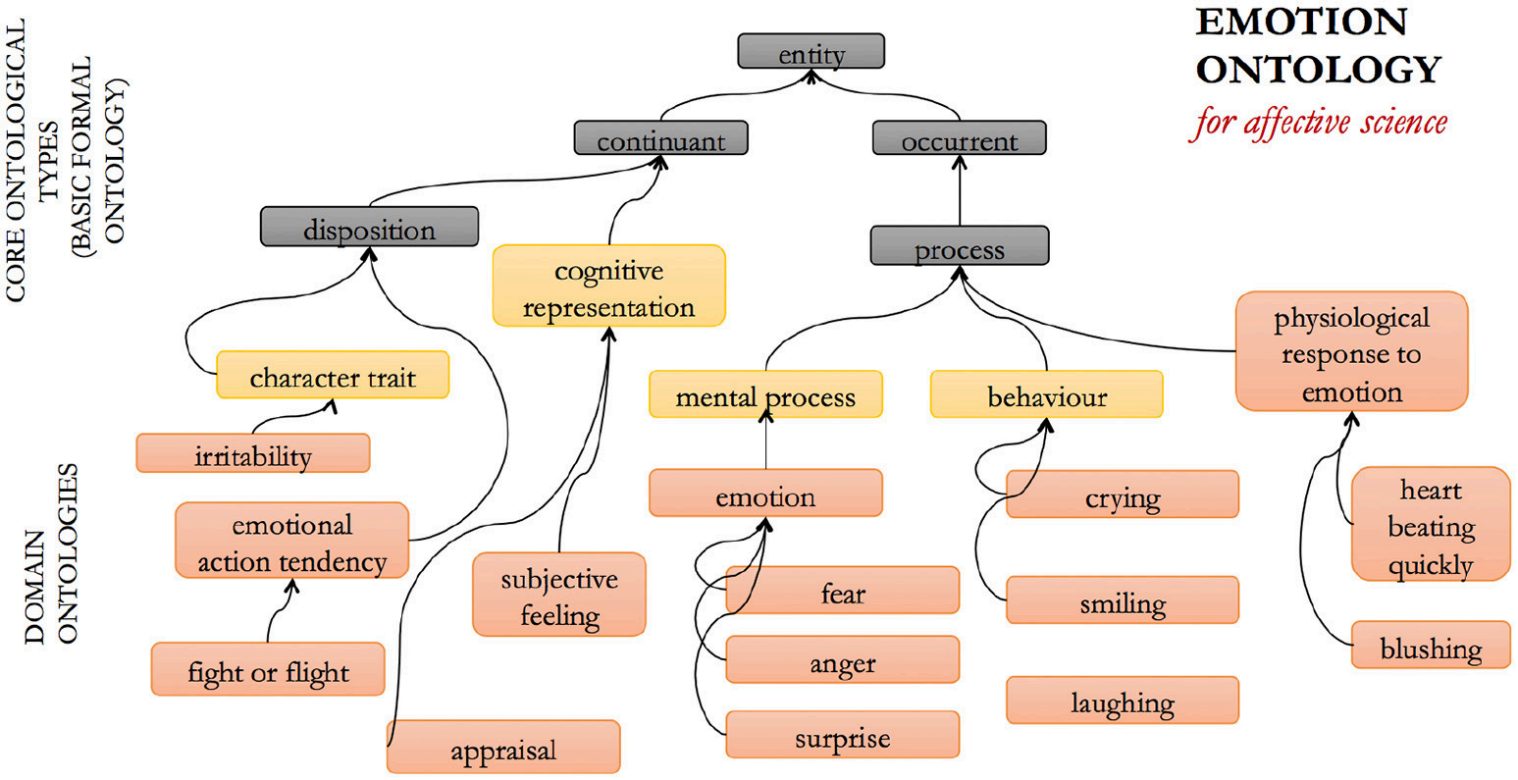
The emotion ontology (EM): selected entities and relations.

The aim of the EM is thus to provide a single ontology, independent of the plurality of theories that researchers have put forward about what emotions are [which are, indeed, many: e.g., (35)]. In this sense, it aims to serve theories of emotion that adhere the James-Lange approach of privileging physiological changes [e.g., (36)] equally as well as, say, appraisal theories of emotion [e.g., (37)]. In order to achieve this objective, a multi-entity framework was developed that explicitly includes different aspects of emotion: the subjective emotional experience, physiological changes accompanying the emotion, the cognitive appraisal that is associated with the emotion, the typical behavioral expression of the emotion including characteristic emotional facial expressions, and so forth. An example of an emotion is “fear,” defined in the EM as “an activated, aversive emotion that motivates attempts to cope with events that provide threats to the survival or well-being of organisms, characterized by feelings of threat and impending doom, and by an urge to get out of the situation” (38). These entities are interrelated and each is specialized into sub-categories. A schematic illustration of some of these entities within the ontology structure is shown in Figure 3 below.

The EM refrains from making claims as to which entities are necessary for valid annotations, and as such, the EM is designed to enable the annotation of maximally complex phenomena, which is partly facilitated by not being committed to any one specific theory of emotion. For example, it leaves open whether the feeling of fear necessarily involves the subjective feeling of fear, and in this way, the EM can accurately annotate situations where a person was frightened, but only realized at a later time that she was in fact frightened. Likewise, the EM can also be used to annotate situations where a person is frightened, yet shows no behavioral expressions.

### 2.3. The mental disease ontology (MD)

**Figure 4:**
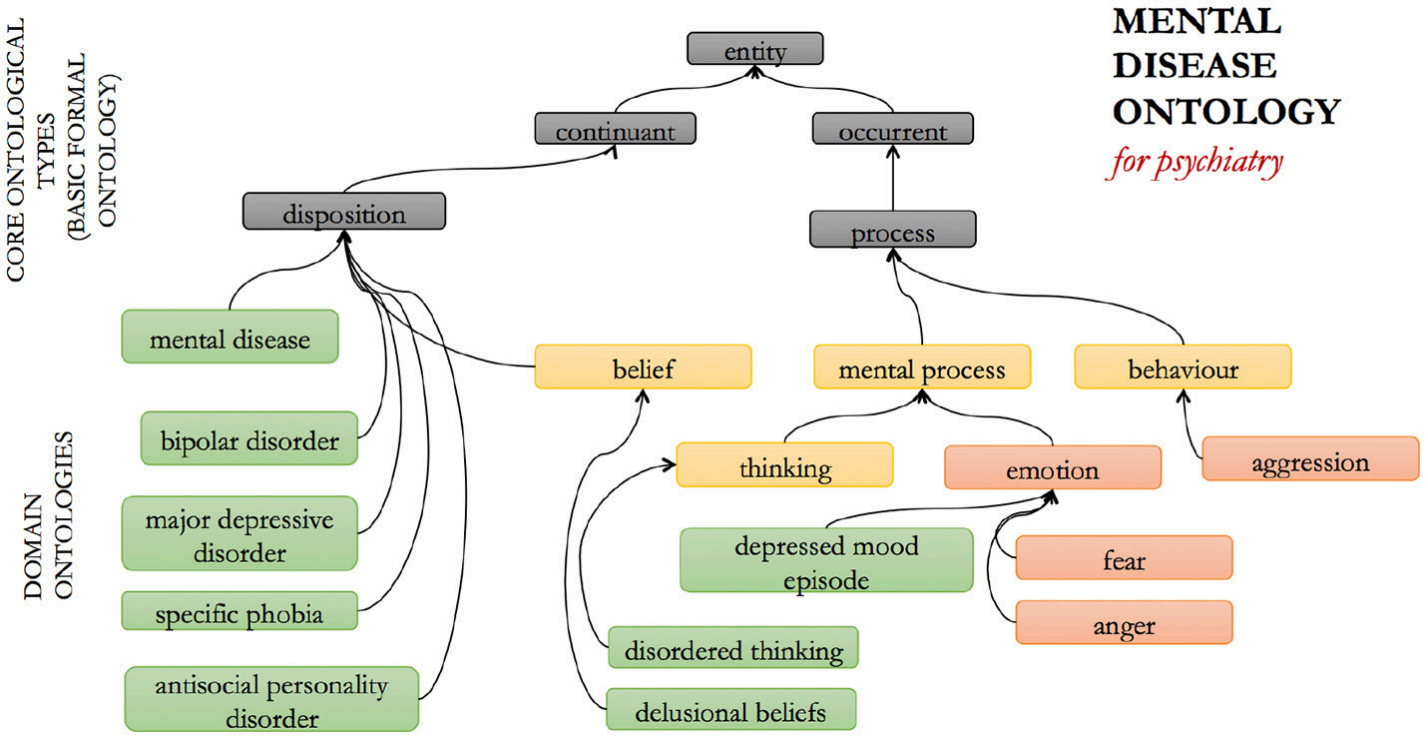
The mental disease (MD) ontology: selected entities and relations.

The MD ontology (Figure 4) was developed in order to provide standardized identifiers for psychiatric disorders across mental health-related data science, i.e., for informed aggregation of data annotations across different branches of research, such as biological, psychological, and psychiatric research (32, 39). Developed on the premise that ontologies should follow community-agreed standards for the nature of the entities delineated in that domain, the MD at present broadly follows the outline of the DSM and ICD approaches, capturing different named disorders for different symptom/sign clusters and organizing them into different groups such as “mood disorders” and “personality disorders.”

It is widely recognized that the DSM as a classification framework suffers from several problems, such as a lack of diagnostic validity, inter-rater reliability, high rates of comorbidity, difficulty in distinguishing “true” cases from false-positive and false-negative instances, over and above the lack of biological markers or specific correlates for specific conditions [e.g., (40)]. Because of these unique challenges, it is often debated whether the DSM categories correspond to “real” diseases or disorders in any meaningful sense, or whether they represent the right way to think about, and structure the research into psychiatric conditions (6).

The framework on which MD was developed assumes that there is some underlying biological correlate for mental dysfunction, which may be considered a somewhat controversial, neo-Kraepelinian assumption [e.g., (41)]. However, the framework does not presuppose the *nature* of the link from measurable physical factors to psychiatric experience: the relationship between physical factors and the various psychiatric conditions is subject to ongoing empirical investigation (42), as is the contribution of cultural and social factors. Facilitating integration of the results arising from such research across different perspectives has the potential to emphasize, rather than hide, relevant historical and cultural contingencies of psychopathological experience, while nevertheless furthering integrative understanding of those aspects of experience that are rooted in specific biological factors. Our assumption that mental health stands in a contingent relationship with physical, measurable factors is equivalent to the observation that we are not dualists, but does not reduce to the claim that all mental diseases are brain diseases *simpliciter*. The MD is not built on the claim that there is a *specific* underlying biological cause for each individual psychiatric disorder type, and that the biological dysfunction is the reason for the development of the psychiatric condition over and above psychological or social factors [for a discussion of this nuance, see (43)]. For most DSM and ICD categories, distinctive underlying etiological processes in this strong sense have yet to be discovered. Rather, we acknowledge the entirety of relevant research, including into the (just as real) socio-historical and cultural factors (42).

Alternative approaches to the DSM are emerging; not only the RDoC framework as already mentioned, but also symptom network (44) and transdiagnostic (45) approaches. The present paper aims to outline an approach that extends the MD, making it fit-for-purpose to support these alternative approaches to thinking about psychiatric disorders and more recent taxonomies for psychopathology, such as HiTOP (18, 19), while at the same time semantically bridging the psychiatric and affective domains.

## 3. Results: semantic bridging

In this section, we outline a broad schematic of ontology entities and candidate relationships for: (a) representing specific affective-related diagnostic entities, i.e., signs and symptoms, in their own right, and linking, that is, *bridging* from those signs and symptoms to traditional disorder categories; (b) showing how a multi-ontology framework with bridging relationships can implement and synthesize the RDoC framework. This schema will make it possible to build complex symptom networks in a shared, ontologically consistent way.

### 3.1. Affective signs and symptoms and DSM categories

**Figure 5:**
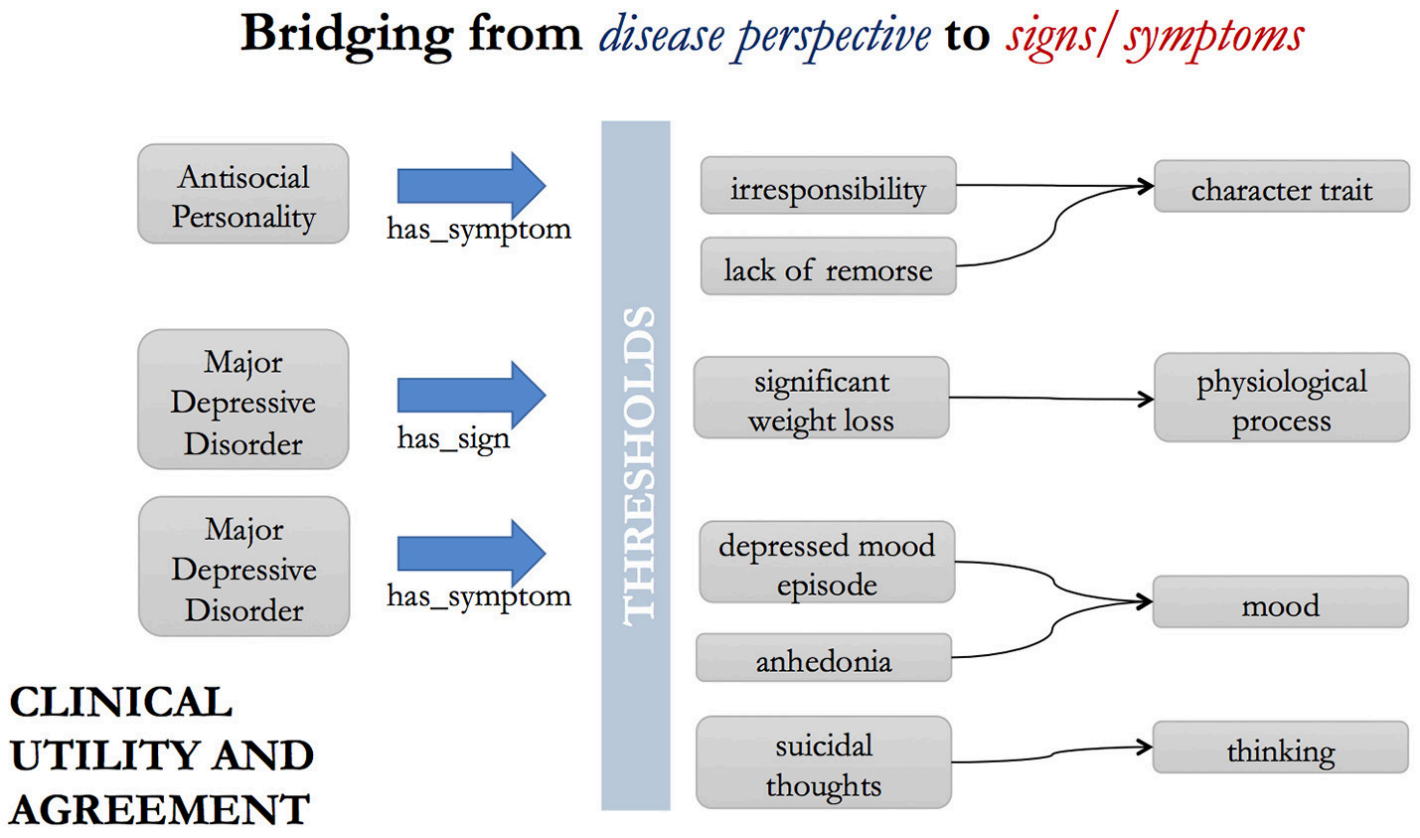
Bridging from the disease/disorder perspective to the sign/symptom perspective.

Psychiatric disorders are typically diagnosed based on the presence or absence of specific signs (i.e., patient behavior) and symptoms (i.e., subjective patient reported experiences). For example, in the case of the DSM diagnostic category Major Depressive Disorder, these include: “suicidal thoughts,” “anhedonia,” “feelings of worthlessness,” “depressed mood lasting longer than 2 weeks” and so on. For another example, say, Antisocial Personality Disorder, the symptoms include “lack of remorse,” “impulsive behavior,” “deceitfulness,” etc. (Figure 5).

A pair of ontology relationships, has_sign and has_symptom, are already used in the Disease Ontology (46), to bridge from disorder to symptom and sign representations.

Thus, we have statements such as:

- “major depressive disorder” has_symptom (“depressed mood episode” that has_duration min “2 weeks”)
- “major depressive disorder” has_sign “significant weight loss when not dieting”
- “major depressive disorder” has_symptom “anhedonia”
- “major depressive disorder” has_symptom “suicidal thoughts”
- “major depressive disorder” has_symptom “loss of energy”
- “major depressive disorder” has_symptom “fatigue”
- “antisocial personality disorder” has_symptom “lack of remorse”
- “antisocial personality disorder” has_symptom “impulsive behavior”
- “antisocial personality disorder” has_symptom “irritability”
- “antisocial personality disorder” has_symptom “irresponsibility.”

The symptoms (depressed mood, suicidal thoughts, etc.) then need to be included in an appropriate taxonomic position within the ontology and given unambiguous definitions in their own right. These symptoms typically do not form a homogeneous group of entities of the same intrinsic class (47). Furthermore, these sorts of entities are not always symptoms of psychiatric disorders, as they may be symptoms of other disorders, or mere instances of innocent and mundane aspects of human mood and affect. For example, “irritability” is a sign/symptom of Antisocial Personality Disorder, but an instance of irritability may alternatively be caused by an external event, or a low blood sugar level. Therefore, we avoid classifying them as subclasses of a single parent class, for example, under the class “psychiatric disorder symptom,” but rather we classify each entity as what they *always* are – moods, emotional episodes, behaviors, etc. The statements above thus act as bridges between the affective sciences and psychiatric science, through the explicit link between psychiatric signs and symptoms that are affective in nature, and the related affective entities themselves. For example, a depressed mood episode will be classified as an affective episode which has as a necessary component “the experience of a deep sadness comparable to grief,” while anhedonia can be defined as an absence or flattening of affect.

One substantial upside of this approach is that it provides a computable and formal representation for the signs and symptoms on their own right, thus supporting annotation of data independent of the traditional diagnostic categories. Such desegregation of data clustering will assist transdiagnostic work, enabling data arising from the isolated investigation of signs and symptoms to be annotated and aggregated across a shared framework.

### 3.2. The RDoC project and a multi-ontology framework

The RDoC Matrix consists of domains or constructs which are intended to be cross-cutting areas of research that have already been identified (by researchers and practitioners) as relevant to psychiatric disorders, such as positive and negative valence systems (i.e., affective phenomena), cognitive systems such as attention, social systems such as attachment and regulatory systems such as arousal and circadian rhythms. Each of these constructs is then laid out across several pre-defined units of analysis, which are as follows: Genes^1^, Molecules, Cells, Circuits, Physiology, Behavior, Self-Report, and Paradigms. Thus, each cell in the RDoC Matrix corresponds to the combination of a unit of analysis and a domain or construct.

Most of the RDoC constructs map straightforwardly onto one of the aspects of “canonical” (i.e., ordinary, non-pathological) mental functioning in the MF or EM ontologies. For instance, MF has perception, attention, language, memory, etc.; EM has fear, anxiety, positive and negative valence, and so forth. “Attachment” is defined in MF beneath “interpersonal process,” alongside “communication,” as is “arousal.”

On the other hand, the RDoC units of analysis, for the biological part, map neatly onto the domains of different ontologies and databases within the OBO Foundry and biological data annotation efforts more broadly. Thus, we have genes which are defined by the various model organism gene building efforts^2^; molecules e.g., proteins in UniProt annotated with the Protein Ontology and smaller molecules in ChEBI; cells which may be described in the cell ontology; circuits and physiological units as defined in various neuroscience resources.

**Figure 6:**
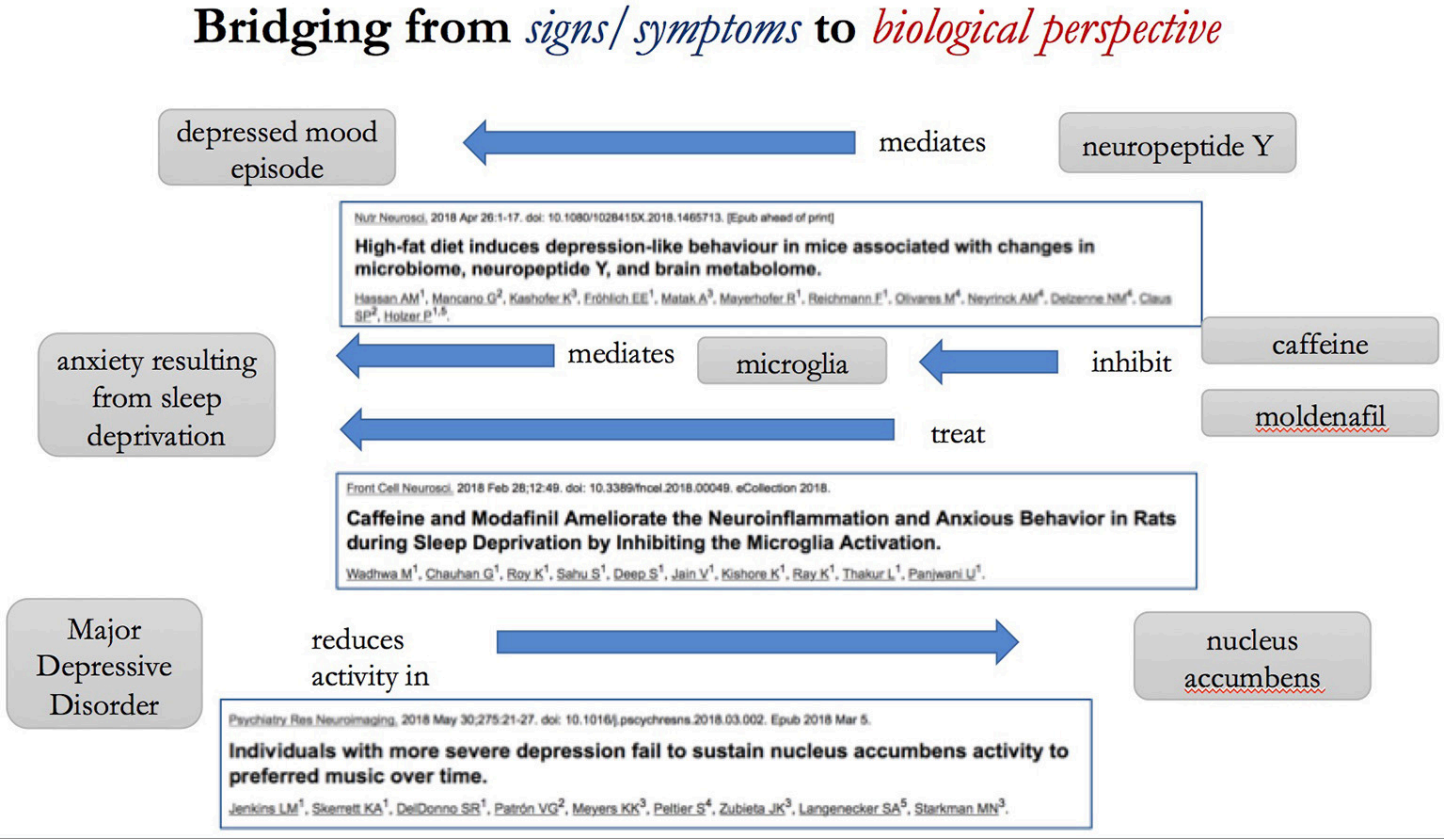
Bridging from the sign/symptom perspective to the biological perspective. The Figure illustrates potential “bridging” annotations taken from recent scientific articles [51–53].

Moving beyond the biological part closer to the psychological part of the RDoC units, the behavior unit maps to the behavior branch of the GO and to the NeuroBehavior Ontology (49), although that ontology focuses mainly on model organisms (e.g., mice) rather than humans who have much more complex behavior. Curiously, some behavioral elements are included in RDoC domains and constructs as well as behavior being listed as a unit of analysis (i.e., behavior is on both “axes” of the RDoC matrix). The further units of analysis are self-reports and paradigms. The self-report unit allows symptoms to be categorized. Paradigms are the stereotypical methodologies used in neuroscientific research with human subjects. The Cognitive Paradigm Ontology (50) includes these sorts of paradigms. Our point is that each of the cells of the RDoC matrix can also be viewed as a bridge – an inter-ontology mapping – between ontologies. Some examples of potential such bridges as drawn from the research literature are shown in Figure 6.

The objectives of the RDoC include many laudable aims to synthesize between and enable knowledge to advance and be translated across the historical boundaries of specific disciplines and methods of investigation. However, it is precisely because the aims of the RDoC are ambitiously multi-disciplinary that no single expert can gather all the relevant information to populate the entire matrix, nor can the bridges between disciplines be captured by any one discipline. What makes ontologies specifically apt to harbor this sort of information is that they are persistent informational entities (similar to databases), cumulative in that their annotations grow over time, and can be contributed to by a full range of community members. As such, ontologies are inherently multi-disciplinary, aiming to describe “what there is” without specifically prejudicing a particular perspective or theory. Their structure is more flexible than a table or matrix, insofar as they (1) provide defined relationships between entities, and (2) are formally structured, enabling the use of logic-based tools to perform automated reasoning (e.g., infer connections that are implied, but not explicitly stated). Similar to the Wikipedia platform for encyclopedic knowledge, the knowledge embodied in the global semantic knowledge base of interconnected ontologies can be informally viewed as the community-wide “hive mind” for scientific entities and their interrelationships.

## 4. Discussion: challenges and limitations

The approach we are proposing entails creating a dynamic, structured knowledge base of the entities that are of relevance across the wide range of topics within the affective and psychiatric sciences, together with associated empirical findings. Definitional and essential knowledge about the field is captured within the (theory-neutral to the largest extent possible) ontological framework, including bridging relationships, while contingent, empirical findings are captured as annotations on entities or relationships between entities. In this fashion, evidence is accumulated at the level of the entity about which an investigation is conducted, and can be synthesized as appropriate to other levels of description, e.g., hierarchically or in support of specific theories.

One possible objection is that we have given too little emphasis to the socio-cultural dimension of mental health, known from epidemiological studies to be of crucial importance to the development of psychiatric conditions, and moreover from comparative and historical analyses to be constitutive of several conditions. We see this issue as orthogonal to, and not incompatible with, our approach. Empirical results arising from social and cultural research can be annotated in our framework in the same fashion, using appropriate classes and relationships, as biological research results. And diagnostic entities that are socio-culturally bound can easily be included in applied ontologies as distinct classes in the framework with their own definitions, as needed to support the annotation of scientific findings. Arguably, some diagnostic entities in the DSM are already of such a type. When enough data has been annotated beneath a shared framework, applied ontologies will be able to assist complex statistical analyses including those that aim to elucidate the mechanisms behind socio-cultural factors in mental health. However, we admit that an acknowledged limitation of our method is that it has limited applicability to data that do not tie in to any empirical research at all.

This approach is of course not a magic bullet that will solve all the theoretical challenges in the field. Many of the challenges that surface in clinics and in laboratories today will still remain even when using the ontological approach. The objective is to enable synthesis of evidence and aggregation of results toward theoretical progress in a more flexible and systematic way than the current methods of labeling and classification allow. In this section, we discuss some of the challenges which our approach faces, which to some extent any labeling and classification approach will face similarly.

### 4.1. Clinical relevance: abnormality and thresholds

One of the core points of contention around the definition and categorisation of psychiatric disorders is the need to delineate between normality and abnormality, that is, delineating why exactly the presence of specific signs and symptoms amounts to a psychiatric diagnosis, i.e., abnormal vs. normal psychological states. For example, an engulfing feeling of sadness and depletion of affective response is considered normal if it is observed in a person who is going through a period of grief, but the same basic feeling can also be a symptom of psychiatric abnormality in Major Depressive Disorder. The original intention of the DSM was not only to bring about a greater standardization in diagnostic terminology, but also to reach a likewise greater agreement among practitioners and researchers in the ways of drawing thresholds in support of clinical practice (6). However, the task of drawing a threshold between normality and abnormality in psychiatry turns out to be more problematic than with typical somatic conditions [e.g., (54)]. A central reason for this difference is that many of todays clinical practice with somatic pathology is grounded in, and supported by a more complete scientific framework and understanding of (what we shall call) canonical biological dispositions and their related processes, compared to the psychiatric domain.

By the term canonical we refer to those biological dispositions that have been thoroughly mapped by the relevant sciences, making it possible to predict specific processes conditioned on said dispositions. Dispositions are potentialities we have in virtue of our physical constitution, and these dispositions are realized in specific processes if certain conditions prevail [e.g., (55)]. For example, human beings are canonically disposed to develop 32 teeth (8 incisors, 4 canines, 8 premolars, 8 molars, and 4 wisdom teeth), and we can thereby assert that it is canonical for the human organism to have 32 teeth. This knowledge is not an outcome of conceptual analysis, but rather acquired through empirical research in human biology (anatomical as well as molecular). One of the central aims in the life sciences is to discover and map these canonical dispositions and their related processes.

Obviously, there are many reasons why humans can end up not realizing their canonical dispositions, for example, not having 32 teeth. But whenever it happens that a canonical disposition has failed to manifest, we know that there must be an etiological explanation for the non-canonical finding; an inference we can draw because we have scientific insight about this aspect of human nature. It is important to emphasize that this perspective on canonical vs. non-canonical is distinctly different from well-known terms in statistics, such as median, mean, mode, range, outliers, etc. While we often speak about what is statistically normal and abnormal, this should not be confused with what we can say is canonical or non-canonical. Indeed, many canonical states will in fact be statistically abnormal, and many statistically normal states can be biologically non-canonical. For example, many people will not have all of their 32 teeth at some point in their lifespan (for various reasons), making it statistically normal to have a non-canonical number of teeth. The point here being that if we did not have the canonical norms available as reference, we would be inclined to think that having 32 teeth was abnormal, while, in fact, it is the other way around.

Determination of abnormality in this sense is still a matter of clinical judgement for psychiatric conditions and usually necessitates drawing thresholds. In practical terms, the thresholds we wish to draw in a clinical setting are based on deciding which phenomena, and to which extent, are clinically relevant. Although there are several ways to approach this, we draw on specific resources used within other applied ontology efforts for the demarcation of these diagnostic thresholds [e.g., (39, 47, 56)].

Most dispositions are realized on a scale. For example, sometimes a situation makes us feel a little low, while other times a similar situations might enormously sadden us. In addition to this, some dispositions also have a normative frame of reference, meaning that specific trigger conditions are expected to realize the disposition in reliable, specific ways. For example, the disposition to feel nausea is reliably triggered by emetic substances (e.g., ipecac syrup or spoiled food). Dispositional normativity, then, refers to the degree of functionality of the disposition given the trigger conditions, i.e., a failure to vomit when drinking ipecac syrup would be a dispositional failure, while immediate nausea followed by purging would be a proper realization.

The DSM appears to embrace this observation, for example, with the grief exclusion criteria in Major Depressive Disorder stating that many of the signs and symptoms associated with the disorder could be appropriate or normal reactions to extraordinary circumstances (e.g., natural disaster, bereavement, etc.). While we might easily agree that it is normal to feel sad in light of bereavement, in the context of psychiatric concerns, it is still a central question to define what degree or level of sadness is a normal reaction.

Drawing these kind of thresholds is not only a task of scientifically profiling the relationship between dispositions, trigger conditions, and realizations, but also of conventionally establishing what researchers, practitioners, and patients (i.e., stakeholders) in a concerted fashion judge to be abnormal. For example, deciding what exact degree of sadness is normal vs. abnormal in the aftermath of a bereavement must, to a considerable degree, involve fiat decisions. Indeed, the diagnostic ideal that all medical abnormalities will be revealed in abnormal test scores, and that normal health will result in likewise normal test scores is an ideal that is seldom met even outside the psychiatric domain [e.g., (57)]. Consider a relatively simple test for hyperglycemia, which measures the concentration of glucose in blood. The diagnostic threshold for fasting glucose levels in blood is set to 100 mg glucose/dL. But this concentration is not necessarily a sign of medical abnormality, as non-diabetic individuals can have these levels. Similarly, diabetic patients may measure below the threshold.

It follows from this that the very same presentations of signs and symptoms may be clinically relevant in one patient and not in another, and that the means and knowledge needed to draw inferences about the underlying canonical dispositions may be entirely or partly lacking, partly due to a still limited science of the enormously complex human organism, and also partly due to our inability to sufficiently isolate human experiences in laboratory contexts. While these are, of course, trivial observations, it is our view that having a framework for data accumulation, annotation, and synthesis that is able to integrate all the relevant information and include a detailed description of the wider context in which the decisions about clinical relevance are taken, will enhance the research efforts that aim to discover and map canonical dispositions in the psychiatric domain.

### 4.2. Partial and incomplete knowledge

It is the nature of the complex human dispositions involved in affect and in psychiatric conditions, that their underlying biological correlates are similarly complex and distributed across many different cells and systems. Therefore, many of the bridging statements that need to be captured in a knowledge framework of the type we describe are only weakly causal: the effect they have in isolation from other causal factors is small. For example, many genes have been implicated in depression, yet none of the relevant alleles have been found to be individually or in concert able to cause depression [see e.g., (58)], and may or may not be present in a particular individual with depression, moreover the magnitude of the influence described may vary from case to case.

In such situations, the imprecise nature of the bridging relationship causes potential problems for its accurate representation in an ontological semantic framework, since the logic on which such a framework is based usually admits only of binary (true or false) interpretations. Our strategy for such representation is to harness the disposition model and thereby represent varieties of “influence” as dispositions that may have an associated strength and conditions for realization (59). In these cases we would annotate, say, a particular gene as having the potential (a disposition) to cause, say, Major Depression, while at the same time reflect that this particular disposition is not very strong. Realizations of dispositions are necessarily contingent on a range of triggering conditions, and the strength of the disposition is reflected in the relationship between the causal factor that bears that disposition and the triggering conditions it requires. Consider as an analogy that both ordinary glass and reinforced windscreen glass have a disposition to shatter, but in the windscreen the disposition to shatter is weaker, thus a stronger force is required to shatter it.

An ontological framework of this kind is able to represent important distinctions in terms of the character or type of influence that are represented by a semantic bridge. Indeed, the influences that might be annotated include not just those that are causally stimulating, but also those that are inhibitory: an entity such as a gene might act as a causal trigger of a condition, or its effect might be to hinder the triggering of a given condition. It may even be the case that one gene can have a triggering effect on the development of depressive disorders, while simultaneously playing an inhibitory role in the development of phobias. Thus, semantic bridges are associated with a hierarchy of relationship types to represent these different types of influence.

The strength of causal connections, and their nature, is only one aspect of the representation of partial and incomplete knowledge. It is also very important to keep track of the epistemological status of a given assertion within a knowledge base, that is, how much evidence we have for that assertion – and how much we trust evidence of that type. Causal factors might have been identified based on population-wide studies or been extrapolated from low-level laboratory experiments in model organisms. The research methodology gives a frame to the type of knowledge that may be discovered and the confidence with which it can be ascribed. Mechanistic details laying out the steps of influence between the relevant biological entity and the affective or psychiatric condition being studied may simply not yet be known.

Semantic bridges, such as the association of a particular gene with a symptom or disorder category, can thus be associated with an evidence code (60) and confidence assertion (61), allowing the resulting knowledge base to be partitioned, if needed, to distinguish between high-confidence and low-confidence findings.

### 4.3. Psychiatric diseases as contested entities

We have claimed above that ontologies allow representation of domain entities in a way that can be neutral with regard to theoretical divisions in a given domain, allowing empirical research results to be accumulated and subsequently evaluated and compared in the context of different theoretical frameworks. Achieving this neutrality, however, is particularly challenging in cases where the entities themselves are only posited to exist within the context of a particular theoretical framework. Psychiatric diagnostic categories (e.g., of the DSM) are contested as bona fide entities, and it is also contested whether these entities correspond to true biological dysfunctions [e.g., (40)].

It is almost universally accepted that psychiatric disorders are not merely brain diseases. They are trivially brain diseases, in the sense that the symptoms are almost exclusively dysfunctions in capacities of the brain, but they are not “simple” or “direct” brain diseases, in the sense that no obvious unitary malfunction in brain cells or neurobiology has yet been found to be the cause in most psychiatric disorders. Rather, it is generally taken to be the case that differences in underlying biology—not themselves necessarily pathological—interact in complex ways with experiences and environment, psychological and social factors, to give rise to the development of a psychiatric disorder in a very individual way for each patient. This is known as the “biopsychosocial” model (62, 63).

The ontological theory of dispositions allows ontologies to capture the complex reality of the biopsychosocial model for mental illness, by distinguishing between the complex dispositions we have based on our physiological makeup and our past experiences, and the realizations or dysfunctions that arise in specific experiences depending on our environment and affordances. To allow the best possible chance of contextualizing the patients experience and finding the real drivers of illness, the biopsychosocial model necessitates that complex clinical, social and psychological histories are taken in order to contextualize any data that arises when studying patients. Standardized questionnaires aim to elicit and record some of this sort of information. Our approach would favor adding each of the entities that feature in such questionnaires to the ontology in its own right, appropriately classified, rather than just the summary outcome, which may be attribution of a diagnosis, corresponding to a potentially contested entity. This may seem as though it will lead to a data explosion, but on the other hand if embedded into the right sort of information system, it will make reporting, as well as comparison and synthesis between studies using different questionnaires, easier in the long run.

What our approach suggests is data annotation to exactly the level that a particular body of research was conducted at, i.e., not necessarily whole syndromes or diagnostic categories but rather the specific symptoms, experiences or behaviors which were the proxy for the diagnostic category in that particular research study. This will enable more informed synthesis between studies, facilitating the harnessing of research results as evidence toward the eventual theoretical progress within the field. It is in line with the proposal of the RDoC to focus on cross-cutting constructs, but allows the domain of discourse to be flexibly defined by the laboratories and clinics in which the research is being conducted.

### 4.4. Clinical information systems: integration and translation

Creating formal connections between diagnoses, symptoms, and other sorts of entities as we have proposed is close in spirit to the approaches which describe individual symptoms and seek to infer from data how those symptoms are related in a network structure (64), and indeed would be compatible with such approaches, but on the other hand while those approaches to some extent disconnect the symptoms they study from traditional diagnoses, our approach would seek to to maintain all entities and associations as separate data annotations.

Our approach by its nature represents a large-scale, complex data annotation and knowledge building effort, far larger than can be conducted by any one person or group. We are proposing bridges between multiple ontologies, each of which is owned and funded through separate pathways. The key to the success of such an endeavor would be community participation in shared distributed knowledge building and annotation activities across multiple different data resources, with the resulting resources being integrated, synthesized, presented and mirrored widely and in open access. This raises well-known challenges around privacy and consent for the use of data with human subjects (65). Furthermore, there are institutional challenges in creating the clinical informatics infrastructure capable of supporting knowledge building and data generation activity with this sort of scope. Our adoption of open source, open access methodology is intended to allow for open participation from across disciplines and locations. We are also able to re-use content from existing databases where those have been similarly openly developed. Text mining of electronic health records is one approach that can help with large-scale automatic data generation [e.g., (66)].

## 5. Conclusions

Bluhm (17) argues that because research progresses by defining constructs “bent toward the laboratory” in different ways for different fields, each inter-disciplinary integration constrains the allowed ontology that can faithfully represent both sets of constructs in the underlying fields. If true, this would lead to an narrowing of the subject matter that worsens with each additional field being integrated in an effort such as that we propose. Bluhm concludes that the development of an integrative ontology would therefore necessitate that the entities within such an ontology have limited applicability in the clinic. Thus, she proposes the development of dual ontologies for different purposes, referring to the research layer ontology as “explanatory” and the clinical layer ontology as “predictive.”

It must follow from this approach—having dual ontologies for these dual purposes within the same domain—that neither of these ontologies are aspiring to become “realist” in the sense of capturing the essence of what exists in the reality beyond the “lenses” afforded by research methods and clinical practice. Yet, most research scientists and clinicians do tend to believe that the targets of their work are the real entities in the world, even though their methods of gaining access to that reality may be constrained by practicalities.

Delineating all the entities that are the subject of research in a domain and specifying interrelationships between them, without specifically privileging one theoretical view as the only truth (although we do offer definitions for theories and views and their corresponding entities, some of which may be contested) offers a network with semantics associated: ontological relationships have a rich variety of semantic types. Entities, too, have types and a rich hierarchy. By remaining agnostic in theoretical divides, semantic bridges can serve an unlimited number of competing perspectives on the nature of the entities within each domain equally well. For example, it can serve to annotate research investigating Autism Spectrum Disorder as a cognitive disorder, as well as those theories that hypothesize it to be an affective disorder.

This form of agnostic annotation is in compliance with the realist project that the applied ontologies in the OBO Foundry adheres to Smith and Ceusters (28), aiming to provide an accurate description of the entities in each of the domains, insofar as is possible within the limitations of current scientific methods. Our approach does not offer a new philosophical or metaphysical contribution. The objective is more pragmatic, and is consistent with different philosophical positions, as is further described in Smith and Ceusters (28). What we offer is an approach by which the practical constraints offered by the methods we have available can be systematized in a framework that allows for needed operationalisations to be captured explicitly alongside the subject matter. When the implicit has been made explicit in this fashion, different results can be synthesized or disentangled as an explicit selection depending on the nature of the question that needs to be answered. It is thus integrative, but not reductive: both the clinical and the research perspective are given appropriate treatment.

## Data availability

The ontology files and all annotations related to this paper can be found in the Mental Functioning Ontology GitHub repository at https://github.com/jannahastings/mental-functioning-ontology/.

## Author contributions

All authors listed have made a substantial, direct and intellectual contribution to the work, and approved it for publication.

### Conflict of interest statement

The authors declare that the research was conducted in the absence of any commercial or financial relationships that could be construed as a potential conflict of interest.

## Footnotes

^1^Note that in May 2017, references to specific genes were removed from the RDoC Matrix following the discrediting of the candidate gene approach in psychiatric research [e.g., (48)].

^2^Note that genes are not defined in the Gene Ontology (GO). The GO describes how and where genes act (their functions and cellular locations), not genes themselves.
